# Missed Opportunities: Refusal to Confirm Reactive Rapid HIV Tests in the Emergency Department

**DOI:** 10.1371/journal.pone.0053408

**Published:** 2013-01-08

**Authors:** Ishani Ganguli, Jamie E. Collins, William M. Reichmann, Elena Losina, Jeffrey N. Katz, Christian Arbelaez, Laurel A. Donnell-Fink, Rochelle P. Walensky

**Affiliations:** 1 Department of Medicine, Massachusetts General Hospital, Boston, Massachusetts, United States of America; 2 Department of Orthopedic Surgery, Brigham and Women’s Hospital, Boston, Massachusetts, United States of America; 3 Department of Biostatistics, Boston University School of Public Health, Boston, Massachusetts, United States of America; 4 Harvard Medical School, Boston, Massachusetts, United States of America; 5 Harvard University Center for AIDS Research, Harvard University, Boston, Massachusetts, United States of America; 6 Division of Rheumatology, Immunology and Allergy, Department of Medicine, Brigham and Women’s Hospital, Boston, Massachusetts, United States of America; 7 Department of Epidemiology, Harvard School of Public Health, Boston, Massachusetts, United States of America; 8 Department of Emergency Medicine, Brigham and Women’s Hospital, Boston, Massachusetts, United States of America; 9 Divisions of Infectious Disease and General Medicine, Department of Medicine, Massachusetts General Hospital, Boston, Massachusetts, United States of America; 10 Division of Infectious Disease, Department of Medicine, Brigham and Women’s Hospital, Boston, Massachusetts, United States of America; Vanderbilt University, United States of America

## Abstract

**Background:**

HIV infection remains a major US public health concern. While HIV-infected individuals now benefit from earlier diagnosis and improved treatment options, progress is tempered by large numbers of newly diagnosed patients who are lost to follow-up prior to disease confirmation and linkage to care.

**Methodology:**

In the randomized, controlled USHER trial, we offered rapid HIV tests to patients presenting to a Boston, MA emergency department. Separate written informed consent was required for confirmatory testing. In a secondary analysis, we compared participants with reactive results who did and did not complete confirmatory testing to identify factors associated with refusal to complete the confirmation protocol.

**Principal Findings:**

Thirteen of 62 (21.0%, 95% CI (11.7%, 33.2%)) participants with reactive rapid HIV tests refused confirmation; women, younger participants, African Americans, and those with fewer HIV risks, with lower income, and without primary care doctors were more likely to refuse. We projected that up to four true HIV cases were lost at the confirmation stage.

**Conclusions:**

These findings underscore the need to better understand the factors associated with refusal to confirm reactive HIV testing and to identify interventions that will facilitate confirmatory testing and linkage to care among these populations.

**Trial Registration:**

ClinicalTrials.gov NCT00502944; NCT01258582.

## Introduction

Expanded HIV screening has led more HIV-infected individuals to be diagnosed earlier in the disease process [1]–[3]. However, the goals of such efforts are often forestalled by continued difficulty in confirming HIV diagnoses and linking patients to care [4], [5].

Following the Center for Disease Control and Prevention’s 2006 guidelines for routine HIV screening [6], numerous reports have documented successful testing programs in the emergency department (ED), one of the guideline-targeted settings. Still, HIV testing remains underutilized, and even when tests are offered, patient refusal is a substantial barrier to optimal screening [7]–[12].

The rapid screening tests frequently used in the ED require Western Blot confirmation of reactive results. This is a critical step in the diagnosis pathway, especially given numerous reports of rapid test false positivity in various settings and in regions such as New York City and San Francisco [13]–[17]. Yet patients are frequently lost at the confirmatory step – a process that requires obtaining consent in some settings, collecting a laboratory sample, and conveying the results [18].This is especially true in fast-paced urgent and emergent care settings [19]. In a screening program at Howard University Hospital, only 39 of 130 patients with reactive rapid tests presented for off-site confirmatory testing [20]. At another urban university hospital ED in Washington, DC, half of the 26 patients with reactive rapid tests were lost to follow-up [21]. Neither these studies, nor others to our knowledge, were able to collect data on why patients did not obtain confirmatory testing.

It is critical to the testing mission to identify the individuals who are more likely to be lost at this first step towards linkage to care. In doing so, we can target efforts to improve rates of confirmatory testing, and in turn, timely treatment. To this end, we conducted a secondary analysis of a large clinical trial – the first to our knowledge – to examine the frequency of failed confirmation as well as demographic variables and HIV risk factors associated with refusal to confirm a reactive rapid test. We then estimated the number of missed opportunities to link to HIV care among those who refused confirmatory testing.

## Methods

### Objectives

Our goal was to determine the rate of refusal to confirm reactive rapid HIV tests in an urban emergency department, to characterize the population who refused confirmation, and to estimate the number of subjects with true positive HIV tests who did not receive confirmatory testing.

### Participants

The study was conducted within the Universal Screening for HIV-infection in the Emergency Room (USHER) trial [22], [23]. USHER was a randomized controlled trial of rapid HIV testing in consenting adults who presented to the ED at Brigham and Women’s Hospital (BWH), a tertiary academic medical center in Boston, Massachusetts. Eligible patients (age 18–75, fluent in English or Spanish, not receiving pre-natal care) were invited by a bilingual counselor to enroll in an HIV testing trial. Patients were consecutively enrolled between 8AM and midnight for at least 60 hours per week. Upon consenting to trial participation, subjects were block randomized by age (<40 years old and ≥40 years old) and sex.

### Description of Investigation

In USHER Phase I (February 7, 2007 to July 9, 2008), enrolled subjects were randomized to oral rapid testing offered by either an ED provider or a dedicated HIV counselor [23]). With permission of the institutional review board (IRB), subjects were enrolled and randomized to testing by an ED provider or HIV counselor after the Phase I stop date and before Phase II was approved (July 10, 2008 to May 1, 2009). In USHER Phase II (May 5, 2009 to January 4, 2010) [22], HIV counselors offered testing to all enrolled subjects who were then randomized by testing modality to either the oral or fingerstick rapid test (OraQuick® ADVANCETM Rapid HIV-1/2 Antibody Test, OraSure Technologies, Inc. Bethlehem, PA). All data from Phase I, Phase II, and the interim period were included in this analysis.

During the informed consent process for the rapid test, subjects were told that “A small proportion of people may have a reactive test. **A REACTIVE** test means you **might** have **HIV infection**. To be sure, a second test must be done. It is important to know that 3 out of 4 patients with a “reactive” test do **NOT** have HIV infection.” After extensive discussion with the IRB, and with the important goal of maintaining the informed consent at or below an eighth grade reading level, we opted not to include additional details about the role of site-specific performance data of the assay or the observed local rate of disease.

Negative results were given to the patient as soon as they became available. Reactive results were given by the HIV counselor or ED provider after the initial medical encounter was complete and a provisional plan had been made to admit, observe, or discharge. This sequence assured that the patient’s chief complaint was fully addressed before introducing the complication of a reactive HIV test; the protocol did not permit discharge with pending HIV test results.

Among patients with reactive test results, early study results indicated that the positive predictive value of the oral rapid test was lower than anticipated. Based on this finding, we hypothesized that predictive value varied by test signal intensity and testers began recording the darkness of the line for reactive rapid tests in both phases of the study [24]. Since rapid test results were maintained outside of the medical record for purposes of the trial, the IRB required that individuals with reactive results provide a second informed consent to complete the HIV confirmatory test panel – Massachusetts law at that time required that this consent be written. Subjects were documented to refuse HIV confirmatory testing if they did not consent to standard ELISA and Western Blot testing when offered by the HIV counselor or ED provider. Upon conclusion of the encounter with the participant, the counselor or provider documented that an HIV test was offered and whether it was accepted or refused.

For those with reactive tests who consented to confirmation, blood was then immediately drawn on-site to perform this panel, which included 1) serum enzyme-linked immunoassay (ELISA), 2) serum Western Blot, 3) CD4 count, and 4) plasma HIV-1 RNA. All subjects with reactive rapid tests were scheduled to receive the results of confirmatory studies within seven to ten days at a follow-up appointment with a hospital infectious disease doctor. In the interim, they were either seen by an HIV social worker in the ED or scheduled to meet with one within two business days (depending on time of presentation and who had administered the rapid test) to facilitate follow-up and provide support.

All subjects with reactive tests were tracked based on whether they were admitted and whether they attended their scheduled appointments with the HIV social worker and HIV care provider. For subjects opting for outside follow-up and confirmation, the research assistant requested release of information and collected the subjects’ contact information and follow-up plans to confirm successful follow-up.

At time of enrollment, the HIV counselor collected demographic data including sex, age, race/ethnicity, primary language, and education level. Subjects were also asked to complete an 86-item self-administered questionnaire, either on paper or by audio computer-assisted self-interview (ACASI) per their preference [25]. This questionnaire collected information on annual household income, medical insurance status, access to a primary care provider, HIV risk factors, HIV-related knowledge, self-perceived need for HIV testing, and HIV testing history. The survey addressed HIV risk behaviors through questions about sexual practices and frequency of illicit drug and alcohol use (Alcohol Use Disorders Identification Test [AUDIT]) [26]. Subjects were risk-categorized based upon previously described definitions of risk [7]: they were classified as having high sexual risk if they reported being a male who has sex with males or noted having more than one sexual partner in the last three months, having sex with a person who was HIV-positive or had AIDS, having sex with someone who has been incarcerated, or using a condom sometimes or never. High alcohol risk was defined as an AUDIT score of >8 [7] and high drug risk was defined as self-report of using a single illicit drug “occasionally” or more often, or using two or more drugs “once” or more often.

Participants were classified as having an HIV risk behavior if they reported high risk in at least one of these three categories (sexual, drug, and alcohol risk). Participants who denied engaging in any of these risk factors were categorized as not having any risk factors. If participants did not provide complete information for all behaviors, those data were considered “missing.”

### Ethics

Written informed consent was received from all participants and all clinical investigation was conducted according to the principles expressed in the Declaration of Helsinki. The USHER trial was approved by the Partners Human Research Committee (protocol 2006P-000136) and overseen by an Institutional Review Board.

### Statistical Methods

A secondary analysis was performed on data from USHER Phases 1 and 2 and the interim phase. The outcome of interest was refusal to confirm a reactive rapid test. The association of demographic variables and HIV risk factors with confirmation refusal was examined in bivariate analyses. Means and standard deviations were calculated for continuous variables and frequencies were calculated for categorical variables. Fisher’s exact test and t-tests were used to compare confirmers and refusers. These comparisons were exploratory in nature so no adjustment was made for multiple comparisons. Due to the small number of refusers, multivariable models were not examined. Therefore, we could not adjust for potential confounders.

Due to high rates of false positivity with the rapid test in the setting of low HIV prevalence at our site and others, we could not assume that all patients with reactive rapid tests were truly HIV infected [13]–[17]. So, we estimated the number of cases of true HIV infection missed among those who refused confirmatory testing. We used two approaches: first, we calculated the proportion of true positives and the associated exact 95% confidence interval among those who did confirm. We used this value to estimate the number of true cases of HIV infection missed in those who failed to confirm. For the second approach, we stratified the sample by intensity of the line for the rapid test (darker than internal control, lighter than internal control, line intensity missing). It has been shown that positive predictive value improves substantially when reactive results are stratified by line intensity [24]. We calculated the proportion of true positives and the associated exact 95% confidence interval among those who confirmed within each group, then estimated the expected number of HIV-infected participants among those who refused confirmatory testing by multiplying the estimated probability of being a true positive within each line intensity group by the number of non-confirmers in each line intensity group.

All analyses were conducted using SAS software version 9.2 (SAS Institute, Cary, NC).

## Results

Between February 7, 2007 and January 4, 2010, 27,441 patients at the BWH ED were screened for enrollment and 8,882 patients were enrolled in USHER-related activities [23]. The most frequently documented reason for ineligibility was age (n = 6,396; 23.3% of all screened). An additional 12.3% of patients were admitted or discharged before USHER enrollment could be completed (n = 3,381).

**Figure 1:**
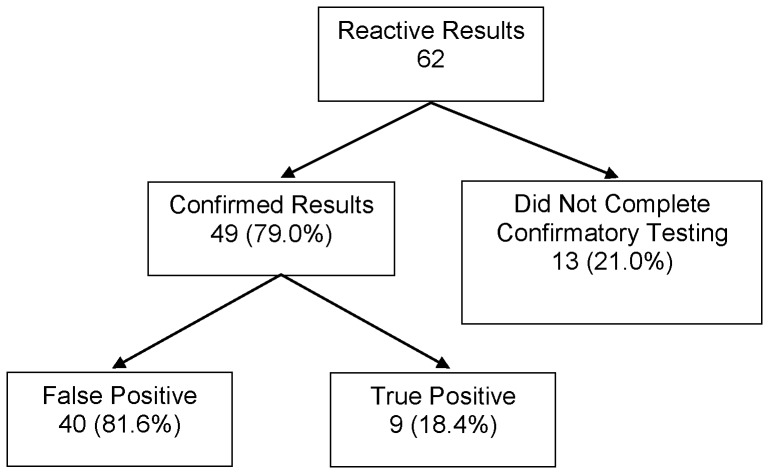
Outcomes of subjects with reactive results on HIV rapid test (1.5% of total tested).

Among 4,056 subjects with valid oral (3,508) or fingerstick (548) test results, 62 had reactive tests (1.5%). Fifty-nine of the subjects with reactive tests received an oral test while three of them received a fingerstick test. Among USHER participants with reactive tests, 49 (79.0%) agreed to confirmatory testing–47 out of 59 (79.7%) of patients who received an oral test and 2 out of 3 (66.7%) patients who received a fingerstick test confirmed. The other 13 subjects refused explicitly. Of the 49 who confirmed, 9 (18.4%) had confirmatory studies indicating true HIV-infection, for an overall undiagnosed HIV prevalence of 9/4,056 or 0.22% (95% CI: 0.10–0.42) (Figure 1). Seven of the nine subjects who confirmed and tested positive attended a follow-up appointment, compared to none of the 13 who refused confirmatory testing.

### Comparing Subjects who did and did not Complete Confirmatory Testing

Subjects who declined confirmatory testing more frequently completed the self-administered questionnaire (12/13, 92.3%) compared to subjects who accepted confirmatory testing (37/49, 75.5%). Among participants with questionnaire data, subjects who declined confirmatory testing were younger on average than those who accepted (40.0 vs 44.9) (Table 1). Females were more likely to decline confirmatory testing (28.6%) than males (11.1%). Among respondents, subjects with incomes greater than $50,000 were less likely to refuse confirmatory testing (15.4%) than those with lower incomes (29.6%) while those with a primary care provider were also less likely to refuse (22.2%) than those without one (33.3%).

**Table 1:** Demographic characteristics of participants with reactive rapid tests who did or did not complete confirmatory testing.

Variable	Subcategory	Confirmed (N = 49)	Unconfirmed (N = 13)	p-value	
Age, years (mean (std dev))		44.9 (14.9)	40.0 (10.4)	0.275	
Sex				0.122	
	Male	24 (88.9%)	3 (11.1%)		
	Female	25 (71.4%)	10 (28.6%)		
Race/Ethnicity				0.127	
	Non-Hispanic White	18 (78.3%)	5 (21.7%)		
	Non-Hispanic Black	12 (63.2%)	7 (36.8%)		
	Hispanic	15 (93.8%)	1 (6.3%)		
	Other	4 (100.0%)	0 (0%)		
Education, more than high school				1.000	
	No	23 (79.3%)	6 (20.7%)		
	Yes	26 (78.8%)	7 (21.2%)		
Income>$50,000				0.741	
	No	19 (70.4%)	8 (29.6%)		
	Yes	11 (84.6%)	2 (15.4%)		
	Missing^a^	7 (77.8%)	2 (22.2%)		
Has a primary care provider				0.649	
	No	4 (66.7%)	2 (33.3%)		
	Yes	28 (77.8%)	8 (22.2%)		
	Missing^a^	5 (71.4%)	2 (28.6%)		
HIV Knowledge, 17 or 18 items correct				0.632	
	No	29 (78.4%)	8 (21.6%)		
	Yes	5 (71.4%)	2 (28.6%)		
	Missing^a^	3 (60.0%)	2 (40.0%)		
Ever been tested for HIV				0.203	
	No	11 (91.7%)	1 (8.3%)		
	Yes	22 (73.3%)	8 (26.7%)		
	Missing^a^	4 (57.1%)	3 (42.9%)		
Perceived need of testing				0.885	
	No	12 (80.0%)	3 (20.0%)		
	Yes	21 (72.4%)	8 (27.6%)		
	Missing^a^	4 (80.0%)	1 (20.0%)		
Sexual Risk				0.392	
	No	4 (80.0%)	1 (20.0%)		
	Yes	27 (79.4%)	7 (20.6%)		
	Missing^a^	6 (60.0%)	4 (40.0%)		
Alcohol Risk				0.062	
	No	30 (81.1%)	7 (18.9%)		
	Yes	3 (100.0%)	0 (0%)		
	Missing^a^	4 (44.4%)	5 (55.6%)		
Illicit Drug Use Risk				0.032	
	No	12 (70.6%)	5 (29.4%)		
	Yes	17 (94.4%)	1 (5.6%)		
	Missing^a^	8 (57.1%)	6 (42.9%)		
Risk Behavior Category				0.058	
	No risk reported	0 (0%)	1 (100.0%)		
	> = 1 Risk reported	31 (81.6%)	7 (18.4%)		
	Missing risk	6 (60.0%)	4 (40.0%)		
	No questionnaire	12 (92.3%)	1 (7.7%)		
Line Intensity				0.298	
	Darker than internal control	7 (63.6%)	4 (36.4%)		
	Lighter than internal control	29 (85.3%)	5 (14.7%)		
	Missing^b^	13 (76.5%)	4 (23.5%)		
Test Type				0.513	
	Fingerstick	2 (66.7%)	1 (33.3%)		
	Oral	47 (79.7%)	20.3%)		

a Excludes participants with no participant questionnaire completed (Twelve subjects in the Confirmed group and one subject in the Unconfirmed group).

b Line intensity was not reported until its correlation with true positivity was suspected. Therefore, the majority of missing data were from early reactive results.

History of prior testing was positively associated with refusal to confirm: of the 30 who reported being previously tested, 8 (26.7%) declined confirmatory testing; and of the 12 who reported never being previously tested, 1 (8.3%) declined confirmation. Subjects in low and high sexual risk categories based on our risk definitions were similarly likely to refuse confirmation (20.0% vs 20.6%). Both low and high risk groups were less likely to refuse than those who did not answer the sexual risk questions (40.0%). 18.9% of subjects in the low alcohol risk category refused confirmatory testing versus none in the high risk category. Subjects in the low drug risk category were also more likely to refuse (29.4%), compared to those in the high risk category (5.6%) – this difference reached statistical significance (p = 0.032).

### Estimating Number of True HIV Cases among those who Refused Confirmatory Testing

Using the proportion of true positives among those who did confirm (18.4%, (95% CI: 8.8% –32.0%)), we estimated that 2.4 of the 13 USHER participants who refused to confirm were likely to be HIV infected (95% CI: 1.1–4.2).

Overall, tests with dark lines were recorded for 11 (17.7%) participants, light lines were recorded for 34 (54.8%) participants, and line intensity was not recorded for 17 participants (27.4%) (Table 2). (Line intensity was not reported until its correlation with true positivity was suspected. Therefore, the majority of missing data were from reactive results found early in the study.) Among subjects who did confirm, those with dark-lined tests were much more likely to be true positives than others: 6/7 subjects with dark-lined tests were true positives (85.7%, 95% CI: (42.1%–99.6%)), 3/13 with line intensity not recorded were true positives (23.1%, 95% CI: (5.0%–53.8%)) and none of the 29 subjects with light-lined tests were true positives (0%, 95% CI: (0%–11.9%)). Among those who refused confirmatory testing, a dark line was recorded for 4 participants, a light line was recorded for 5 participants, and line intensity was missing for 4 participants. By multiplying the likelihood of true positivity in each group by the number of non-confirmers in that group, we predicted that 4.4 (95% CI: 1.9–6.7) of the 13 USHER participants who refused to confirm were likely to be HIV infected.

**Table 2:** Estimating the number of true HIV cases missed.

	Confirmers		Non-Confirmers	
Line Intensity	Total (N)	% True Positive (95% CI)	Total (N)	Estimated # of true positives (95% CI)
Darker than internal control	7	85.7% (42.1%–99.6%)	4	3.4 (1.7–4.0)
Lighter than internal control	29	0% (0%–11.9%)	5	0 (0–0.6)
Missing	13	23.1% (5.0%–53.8%)	4	0.9 (0.2–2.2)
**Total**			**13**	**4.4 (1.9–6.7)**

## Discussion

Among the 4,056 patients who received HIV rapid tests with valid results in the USHER trial, 62 had reactive results. Of these, 49 had confirmatory testing and nine in this group were found to be true positive. The remaining 13 refused confirmatory testing. Women, African Americans, and those self-reporting less HIV risk were more likely to refuse confirmation. We estimated that, depending on the prediction methods used, two to four additional cases of true HIV would have been identified if those 13 participants had agreed to confirmatory testing.

In 2006, the CDC issued guidelines for routine HIV testing with the critical goals to identify and counsel more individuals with unrecognized HIV infection and to link them to care [6]. However, as the USHER trial experience demonstrates, barriers to this goal remain, even once the initial screening test is offered and performed.

In some respects, refusal of confirmatory testing is a similar phenomenon to refusal of the initial screening test. Indeed, the demographic and risk profile of non-confirmers reported here parallels others’ findings on individuals who refuse HIV screening in general [27], [28]. Previously published multivariate analyses from the USHER trial [7] and elsewhere [29] have shown that women and patients with annual household incomes of >$50,000 are more likely to refuse testing, as are individuals who report not engaging in HIV risk behaviors, those who have been tested previously, and those who do not perceive a need for testing. In a series of in-depth interviews with patients who refused HIV testing in the ED setting, cited reasons included: a recent prior test, the perception of low risk, and concerns about distraction from their chief complaint, about the implications for a relationship, and about the documentation of positive HIV status [30].

The decision to confirm a reactive screening test is a distinct phenomenon that incurs the psychological burden of being one step closer to a potential new HIV diagnosis. A key question is the degree to which this burden affects patients’ decisions not to confirm the initial screening result. Individuals who refuse at this stage may be troubled by the implications of a true HIV diagnosis and seek escape. This is corroborated by earlier estimates, prior to scaling up testing efforts, of an 8.1 year lag between acquiring HIV and initial presentation to care [31].

In our study, women and African Americans were less likely to seek confirmatory testing. Though some women may refuse due to their perception of low risk, women are in fact a significant percentage of the U.S. HIV-infected population (25% of those living with HIV infection in 2008) [32], [33], and African American females in particular have disproportionate rates of new infection [33]. Women in particular, in the absence of pregnancy, may be more likely to refuse confirmatory testing because of concerns about stigma and fear of violence [34]. Women may also resist confirmatory testing because subsequent care might compete with other needs such as childcare and personal subsistence [34], [35]. In one study, white women were the most likely not to seek care for an HIV diagnosis in a timely fashion, followed by minority women [36].

All patients may be concerned about having a positive HIV status recorded in their medical charts (this documentation may not occur with a reactive rapid test alone) and the implications for health insurance and employment status. African American patients in particular have been reported to distrust the medical system, particularly when the consequences of the confirmatory testing may have long-term implications [34].

Some participants may have declined confirmatory testing because they worried about accessing follow-up care. Those who reported having a primary care provider were somewhat less likely to refuse confirmatory testing (22.2%) than those without a primary care provider (33.3%). However, 8 out of 12 non-confirmers who responded to our survey noted that they had a primary care physician, suggesting that this does not guarantee easy or affordable access to the expensive anti-retroviral drugs needed for life-sustaining care and further emphasizing the importance of parallel HIV screening efforts in the primary care setting [37]. Subjects reporting incomes greater than $50,000 were more likely to confirm reactive results than those with lower incomes; perhaps subjects with higher incomes felt better-equipped to handle the repercussions of a true positive test.

In the USHER trial, subjects who had previously been tested for HIV were more likely to refuse HIV rapid testing than those who had not previously been tested [7]. Similarly, in our analysis, among the subjects who had a reactive rapid test, those who had been previously tested were also more likely to refuse confirmatory testing, perhaps because they felt reassured by a prior negative test.

Patients may also decline confirmatory testing because of its inconvenience and their desire to leave the ED. Some studies document that ED patients felt that rapid testing did not delay their medical care (92.5%) or divert attention from the reason for their ED visit (94.1%) [38]. However, respondents may have overestimated their acceptance of this confirmatory test, under the assumption that they would not require it. It is also possible that they refused based on knowledge of the oral rapid test’s low specificity: Fifty-nine out of 62 individuals with reactive tests had received an oral test, and they learned in the informed consent process that 3 out of 4 reactive oral rapid tests are false positives. But despite the low specificity of the oral test, a reactive oral test still increases the pre-test probability of infection prior to confirmatory testing by around 300 fold (from 0.078% to 25%), certainly enough to merit follow-up testing [39], [40].

In USHER, most subjects with reactive rapid HIV tests and positive confirmatory testing (7/9) attended their first follow-up appointment as scheduled by the USHER team. Unfortunately, all participants refused requests to inquire about confirmatory results outside the study context (e.g. by calling their primary care providers or by contacting other potential sources of confirmation). As such, we were not able to determine if these subjects sought this testing or care elsewhere.

Of note, subjects who refused confirmatory testing were more likely to agree to complete the 86-item questionnaire compared to those who accepted confirmatory testing (92.3% vs 75.5%). This, and the fact that all subjects in the reactive test group, by definition, agreed to initial testing, suggests that subjects did not refuse confirmatory testing simply because they did not want to participate in research. Rather, they more likely refused due to anxiety surrounding a possible diagnosis or another factor specific to the confirmatory process. We cannot rule out, however, that the Massachusetts requirement for written, rather than verbal, consent at each step did not have an impact on subjects’ willingness to undergo confirmatory testing; we believe that passage of the 2012 Massachusetts law allowing for verbal consent may facilitate consenting efforts.

In compliance with the IRB, the USHER trial required patients to provide separate consent for confirmatory testing, unlike other testing programs which are often able to obtain consent for rapid and confirmatory testing at the same time. The second consent hurdle allowed us a rare look at the most proximal point in linkage to care and to identify individuals most likely to be left behind at this important step. Of note, some EDs have bypassed this step entirely by using either sequential rapid tests [19] or rapid testing on venipuncture specimens, which allow for confirmatory testing on the same sample [41]. Based on the predictive model, two to four true diagnoses of HIV were lost to follow-up. These additional diagnoses would have increased our total number of true positives by up to 49% (4.4/9). This finding, and our characterization of non-confirmers based on demographics, risk factors, and HIV knowledge, underscores the importance of targeting these groups in the testing process.

### Limitations

Results of this study should be interpreted within the context of its limitations. This secondary analysis included a small number of patients with reactive rapid tests, which reflects the relatively low prevalence of HIV and increasing frequency of testing in the Boston area. As a result, we had limited power to detect statistically significant differences between confirmers and refusers (Table 1). The high false positive rates associated with oral rapid testing in the study have been reported elsewhere, in the context of the USHER trial and others [15]; the rapid test manufacturer has since improved the test’s specificity, so confirmatory testing may have been viewed more favorably by subjects if the study had been performed more recently [42]. In the consent process, we did not routinely explain the impact of site-specific assay performance and disease prevalence on the rapid test’s positive predictive value.

In addition, thirteen of the sixty-two participants (21%) refused the questionnaire; among those who completed it, many omitted specific questions. Thus, our capacity to address all of the behavioral associations with refusal was limited. Additionally, if the participants who refused the questionnaire or omitted specific questions are different than those who answered the questions, specifically if non-response is related to refusal to confirm, our results could be biased. Furthermore, our paper is limited to demographic and risk factor description of those who refused to confirm; we were unable to obtain direct data as to why participants refused. However, considering the sensitive nature of the questionnaire content, the relatively low prevalence of HIV, and the difficulty in connecting with the population under consideration, we had greater success than has been previously reported. Further studies should be performed to better characterize why certain subjects may not confirm reactive rapid HIV tests or seek follow-up care.

We estimated the number of cases of true HIV missed in those refusing confirmatory testing by two methods, the first of which used the proportion of true positives in those that did confirm. This method assumed that the rate of true HIV infection is similar in the confirmers and non-confirmer groups and therefore may have produced a conservative estimate, as the non-confirmer group likely had a higher rate of true infection. The small number of cases limited our ability to explore multivariable models to predict the number of cases missed.

In this study, we found that younger subjects, women, African Americans, and those from lower self-reported HIV risk categories, with lower incomes, and without primary care providers were more likely to disengage from the linkage to care process by refusing confirmatory testing. We predicted that two to four cases of true HIV were not diagnosed as a result of refused confirmatory testing. These findings suggest the need for further characterization of at-risk populations and interventions targeting these populations in order to strengthen the very first link in the chain of effective HIV care.
